# Research Trends in Emerging Contaminants on the Aquatic Environments of Tanzania

**DOI:** 10.1155/2016/3769690

**Published:** 2016-02-22

**Authors:** H. Miraji, O. C. Othman, F. N. Ngassapa, E. W. Mureithi

**Affiliations:** ^1^Chemistry Department, School of Physical Sciences, College of Natural and Mathematical Sciences, University of Dodoma, P.O. Box 338, Dodoma, Tanzania; ^2^Chemistry Department, College of Natural and Applied Sciences, University of Dar es Salaam, Dar es Salaam, Tanzania; ^3^Mathematics Department, College of Natural and Applied Sciences, University of Dar es Salaam, Dar es Salaam, Tanzania

## Abstract

The continuity for discovery and production of new chemicals, allied products, and uses has currently resulted into generation of recent form of contaminants known as Emerging Contaminants (ECs). Once in the aquatic environment ECs are carcinogenic and cause other threats to both human's and animals' health. Due to their effects this study was aimed at investigating research trends of ECs in Tanzania. Findings revealed that USA and EU countries were leading in ECs researches, little followed by Asia, South Africa, and then Zambia. Only few guidelines from USA-EPA, WHO, Canada, and Australia existed. Neither published guidelines nor regulations for ECs existed in Tanzania; rather only the occurrence of some disinfection by-products and antibiotics was, respectively, reported in Arusha and Dar es Salaam, Tanzania. As these reports had a limited coverage of ECs, henceforth, these findings constitute the first-line reference materials for ECs research in Tanzania which shall be useful for future monitoring and regulation planning.

## 1. Introduction

Environmental pollution started since the ancient times with the main focus on the regulated pollutants that imposed health and ecological effects. In early 1800s new kind of pollutant named Emerging Contaminants (ECs) was discovered in water and aquatic environments due to development of science and technology [1]. These contaminants are not termed as emerging because of being new in terms of discovery, use of new advanced detection, and treatment methods but because they were previously unrecognized and lacked standards and guidelines for their environmental monitoring and recently they have gained scientific attention because of their health effects [2]. As reported by [3] and [4] nanogram per liter (ng/L) concentration of ECs can exhibit relative effects to both human and aquatic organisms. Some worth mentioning effects of ECs include hormonal interference in fishes, genotoxicity, carcinogenicity in lab animals, endocrine disruption, and immune toxicity [5]. Examples of ECs are pharmaceuticals, cosmetics, endocrine disrupting hormones, disinfection by-products, illicit drugs, nanomaterials, persisting organic compounds, and algal toxins [6, 7].

Since the discovery of ECs around 1970s in USA, four lists of ECs have been published, respectively, in 1998, 2005, 2009, and 2015 [8–10]. The leading countries in ECs research are USA, China, Canada, Germany, and Japan, while Africa is still far back [11]. Absence of guidelines, monitoring regulation, and research activities in Tanzania does not imply nonexistence of ECs [12]. Subsequently all sources of ECs including military sites, mining tailings, agricultural fields, industrial units, waste treatment plants, and pharmaceutical, clinical, and municipal wastewaters prevail in our environment; therefore immediate research on ECs is inevitable. Research in this field is urgent because such data is necessary and more should be known about public health effects particularly in our country. Findings upon occurrence of ECs will be a milestone for public awareness, initiation of control, and monitoring plans as well as an initiation towards mitigation approaches.

## 2. Conceptualization of Emerging Contaminants

All legally synthetic chemicals are monitored by local and international standards for environmental protection. Chemical products are formulated for human, animals, and plants uses. Nevertheless once released in the environment they undergo bioaccumulation, bioconcentration, and persistence in the aquatic environment [13, 14]. Although there are no fully established monitoring regulations their availability brings potential unclear health and ecological risks [15–20]. Most existing studies revealed health effects in laboratory animals, meaning that short or long time exposure is threat to human being [17, 21–24].

## 3. Characteristics and Classification of ECs

Once in the environment ECs are more polar, acidic, and alkaline than natural chemicals making them fetal at minute concentrations. They are hydrophobic which makes them accumulate in the lipid-rich tissues thus being dynamic through food chain [11]. Their low concentrations result in difficulties upon detection and removal from the environment [25]. Emerging Contaminants have generally been quantified by GC/MS and/or GC/MS/MS after solid phase extraction. Raghav et al., 2013 [26] classified ECs with respect to suspected health effects. A more comprehensive and supported approach is by grouping them based on the sources, the effects, the uses, and their chemistry [27–30]. Although there are about 104 ECs, only few classes are discussed as they are likely to occur in Tanzania.

### 3.1. Pharmaceuticals and Endocrine Disrupting Hormones

Pharmaceuticals include prescribed drugs, veterinary drugs, diagnostics agents, and vitamins which are either synthetic or natural medicine. They are used for alteration of physiology and biochemical processes in human and animals for treatment, diagnosis, or prevention of diseases. Their occurrence in the fresh, ground, wastewaters, and aquatic areas is an obvious indication of contamination either through direct disposal of expired/unwanted medicines in the toilets, landfills, and household wastes or through body excretions.  Table 1 indicates selected classes and uses [31], quantitative and qualitative amounts of pharmaceuticals, and endocrine disrupting hormones observed and reported from various published articles. In the aquatic environment they induce antibiotic resistance to disease causing organisms and increase cancer rates and organ damage [32, 33]. Endocrine disrupting hormones include estrone and estriol, impacting and affecting hormonal control and sexual and reproductive behavior [34] while nonprescribed drugs like caffeine, marijuana, cocaine, and cannabis effects are less known they presumed being toxic to aquatics [26, 35]. Physical effects of drugs abusing exist in various parts of Tanzania; unfortunately their environmental consequences are yet to be reported. Similarly applied to pharmaceutical pollution in Tanzania, very limited published information do exist [36].

### 3.2. Personal Care Products (PCPs)

Sunscreen/UV filters found in personal care products act by absorbing, reflecting, and scattering UV light. Some examples of personal care products include masks, siloxanes, benzophenone-3, and octyldimethyl-p-aminobenzoic acid and disinfection products like triclosan. They are used in cosmetics, body sprays, lipsticks, and varieties of home products. Through washing, bathing, swimming, and domestic wastes they get into aquatic environment leading to endocrine disruption, genotoxicity, cytotoxicity, and carcinogenicity [29]. Apart from all these effects yet Tanzania has no public environmental guideline for PCPs pollution, rather it has guidelines for cosmetics and medicines disposal [37]. Significant amounts of UV filters (19 *μ*g/L) and 125 ng/L of benzophenone-3 were reported from Swiss, 10 ng/L of sunscreen was reported from Greece [38]. Full scan PCPs were reported ranging between 5 and 5154 ng/L from Spain [39] and 0.03 *μ*g/L of triclosan from Zambia [40] and 0.43 *μ*g/L of triclosan from Poland [2]. Eventually one country from Africa has reported PCPs (Zambia) while this keyword still seems to be a terminology in Tanzania.

### 3.3. Artificial Sweeteners (ASs)

Commercially artificial sweeteners are added into drinks, foods, drugs, and hygienic products to increase taste with less caloric content. Examples include aspartame, cyclamate, saccharin, stevia, and sucralose. They are generally used for weight loss, dental care, diabetes mellitus control, reactive hypoglycemia, and cost effectiveness. Their presence in the environment is a clear indication for wastewater contamination into surface and groundwater [41]. Adverse effects associated with artificial sweeteners pollution are cancer risks, dysbiosis, glucose intolerance in human health, and weight gaining in children [42–44]. Tran et al., 2014 [41], reported from Singapore the occurrence of ASs in the range of 47–1640 ng/L and 610–3200 ng/L in Germany and 2800–6800 ng/L in Switzerland [41]. About 12.9 *μ*g/L of sucralose was reported from EU countries [45]. Looking in the reported areas mostly Europe, America, and little of Asia frequently appeared while the rest of the world is still silent. This may be justified by very low detection limit of artificial sweeteners (50 ng/L), which requires very advanced technology. Similarly, Tanzania Food and Drug Authority have yet publicized environmental standards and guidelines for artificial sweeteners pollution as per available online information.

### 3.4. Disinfection By-Products (DBPs)

Disinfection agents like chlorine and chloramine are strong oxidizing agents used during the course of water treatment. Their modes of action involve destruction of pathogenic microorganisms and oxidation of taste and odour forming compounds. This action forms disinfectant residual which prevents further growth or contamination by microbes along the way. Reaction between disinfecting chemicals with natural fluvic acid, humic acid, amino acids, and iodide and bromide ions produces DBPs like trihalomethanes, THMs (bromodichloromethane, bromoform, dibromochloromethane, and chloroform), haloacetic acids, HAAs (chloroacetic acid, dichloroacetic acid, trichloroacetic acid, bromoacetic acid, and dibromoacetic acid), chlorates, and bromates [50]. Other DBPs include bromonitromethanes and nitrosodimethylamine (NDMA). Brominated disinfectants are more toxic than chlorinated ones, thus favoring use of chlorinated disinfectants particularly chloroamines [51]. Apart from direct ingestion, showering and dermal absorption are the roots through which DBPs can get into human. A long time exposure has been associated with genotoxicity and carcinogenic effects. Following the discovery of DBPs Canadian standards allow a maximum of 100 *μ*g/L and WHO, respectively, accepts maximum of 60, 100, 100, and 200 *μ*g/L of chloroform, bromodichloromethane, dibromochloromethane, and bromoform. The highest amounts of THMs and NDMA quantified in Canada were 200 *μ*g/L and 180 ng/L [18]. About 50 *μ*g/L of THMs was reported from Zambia [40] and a range of 1.2–97.6 *μ*g/L of DBPs was reported in Arusha Tanzania [52], both of them being within accepted standards.

### 3.5. Perfluorinated Surfactants and Polybrominated Diphenyl Ethers

Perfluorooctane sulfonate (PFOS) and perfluorooctanoic acid (PFOA) are two main representatives of polyfluorinated synthetic chemical which are stable and persistent. They have hydrophobic alkyl chain and hydrophilic functional groups making them useful as detergent, impregnation agent in textile, leather, carpets, firefighting form, cosmetics, and many other consumer products. PFOS and PFOA are of great concern because they are persistent in the environment; they are toxic and they undergo bioaccumulation in biological systems [53, 54]. PFOS and PFOA are broadly used as fire retardant in plastics, textile coatings, and electronic appliances. Their mode of action in different materials involves reducing flammability of fuel and/or delay combustion. It is worth mentioning that fire retardant additives are penta-, octa-, and decabromodiphenyl ether mixtures [55]. They have been observed in the treated waters, sewages, biota, and human body despite their roots being unclear. Flame retardants are lipophilic kind of organic pollutant, less soluble in water, less volatile, and environmental persistent. They are presumed to cause neurotoxicity, hormonal disruptions, and cancerous scenarios [28, 54]. Intrinsic amounts of perfluorinated surfactants, 0.08–0.87 ng/g (ww) in French, 0.04–0.08 in Asia and Brazil, 2.08 ng/g in Korea, and 0.11–0.13 in China and Japan, were reported [56]. Fawell and Ong, 2012, reported a concentration range of 0.12–0.92 ng/L in China [29], 1290–2440 *μ*g/L from human blood in USA, 950–1930 *μ*g/L in Belgium, and 7–40 ng/L in German and 31 *μ*g/L in Norwegian serum [53]. Similar study by Houtman, 2010, reported a noteworthy range of 0.001–1 *μ*g/L from surface waters in Europe [49]. Considerable amounts of polybrominated diphenyl ethers (PBDEs) (7.1–53 pg/m^3^) were detected from air and sewage sludge in Japan and Sweden, 1.23 to 57.2 ng/g was observed from lipid in Europe [38], and 165 ng/L was observed from river water in China [27]. Also PBDEs have been quantified in pg/L in Michigan (USA) and Singapore [18]. Besides these findings Tanzania Bureau of Standards has yet reported primary guidelines and standards for monitoring perfluorinated surfactants and polybrominated diphenyl ethers. In addition there are no published reports on the occurrence of PFOS and PFOA in Tanzania.

### 3.6. Benzotriazoles and Naphthenic Acids

Benzotriazole and tolyltriazole are anticorrosive agents used industrially and at household scales. They occur in surface and sewages, characterized by high water solubility, photosensitivity, and resistance to biodegradation. Naphthenic acid is mixture of several cyclohexyl and cyclopentyl carboxylic acids naturally found in petroleum, oil sands, bitumen, and crude oils. Main uses include wood perseveration and paint additive. Primary root in the environment is mostly through discharges while coastal erosions and groundwater mixing have insignificant contribution. Environmental persistence caused by its weak degradation, corrosion properties, and tailing concentrations of naphthenic acid pose great analytical challenges [57, 58]. Nevertheless, there were no existing data from Tanzania Bureau of Standards, contaminant candidate list 3, and EPA regarding maximum accepted levels of benzotriazoles and naphthenic acids. Moreover, research information regarding aquatic pollution caused by naphthenic acids is scarce. While reports and guidelines for benzotriazoles and naphthenic acid are yet published in Tanzania, considerable amounts (550–1380 ng/L) of benzotriazole in the wastewater treatment plants effluents were reported in China. This amount was lower than 17–44 *μ*g/L reported form Berlin [20] and 7.27 *μ*g/L observed in Greece [59]. The highest amount of benzotriazole, 221 *μ*g/L, was detected in Italy [45].

### 3.7. Algal Toxins Produced by Blue-Green Algae

Blue-green algae also called cyanobacteria naturally occur as photosynthetic simple plants in nonturbulent surface waters. Its ability of fixing nitrogen and vertical migration in water column makes them competitive edges of aquatic ecosystems. Nutrients rich waste discharges in aquatic environment cause algal blooms. Blue-green alga produces toxins such as microcystin, anatoxin, and saxitoxins. Their toxins target nervous system, brain, and liver meaning that at very low concentrations toddlers and elders are more vulnerable. Also algal toxins were suspected to cause paralytic shellfish poisoning. It was reported from USA that approximately 50–75 *μ*g microcystin/L intoxicate aquatic organisms may kill fish if exposed for 24 hours [60, 61]. Algal bloom has been reported in Tanzania in the aspects of water transparence, photosynthesis, and oxygen supply [62]. Australian government set 1.3 *μ*g/L of microcystin as the limit in drinking water [38]. However, standards, guidelines, and research activities for algal toxins are yet in place in Tanzania.

### 3.8. Perchlorate

Environmental occurrence of perchlorate is both natural and man-made. It is used in energetic boosters such as rocket fuel, fireworks, flame, missiles, explosive, and fertilizers. The fewer existing reports show that abundance in the sewages, natural waters, and aquifers appears as a contaminant. In the environment perchlorate tends to be highly soluble in water, relatively stable, and not volatile. Short time exposure causes eye, skin, and respiratory tract irritation, coughing, nausea, vomiting, and diarrhea [63]. Perchlorate chemical contaminant can disrupt thyroid ability to produce hormones. It was reported by Urbansky and Schock, 1999 [64], that EPA has set perchlorate risk level of 5 ng/L that was considered being below the detection limit of ion chromatography. Owing to Environmental Protection Agency standards, only less than 2.5 ng/L of perchlorate is allowed in the water supply. Parker, 2009, reported an average of perchlorate concentration of about 2 *μ*g/L in New York [65]. In fact quantification of perchlorate to these levels presents significant technological challenges in Tanzania. Besides published reports and standards from USA, there were no immediate publication, guidelines, or perchlorate standards in Tanzania.

## 4. Conclusion

Tanzania is still combating communicable and noncommunicable diseases like malaria, respiratory, skin, and eye infections. Also major cities of Tanzania experience intensive stress on construction, industries, and rapid population growth. In addition, several media have reported periodic destruction/disposal of out-of-order cosmetics, drugs, and electronics. Therefore, the proof for prevalence of ECs in Tanzania exists; however, it was observed that there was no research coverage in ECs field, rather at least disinfection by-products and antibiotics were reported. Beyond this neither immediate published reports nor guidelines do existed. Hence these findings are milestone for scientific, managerial, and political call in this field in order to pace as the world step.

## Figures and Tables

**Table 1:** Pharmaceuticals and endocrine disrupting hormones.

Class	Drugs/hormones	Reported amounts (*μ*g/L)/country	References
Antipyretics for reducing fever	Aspirin	0.22 (Germany), 13 (Greek, Spain)	[46]
	Naproxen	<0.1 (USA), 0.958 (EU), 0.108 (Spain), 0.7 (Poland)	[2, 45, 47]
Analgesics for pain relief	Paracetamol/acetaminophen	0.211 (USA), 10 (USA), 10.19 (Spain)	[16, 18, 47]
	Ibuprofen	516 (USA), 0.174 (China), 2.5 (Poland), 70.35 (USA), 6.0 (Spain)	[2, 30, 39, 47, 48]
Antibiotic for treating bacterial infections	Metronidazole	0.176 (Spain), 0.9 (Switzerland)	[20, 39]
	Tetracycline	0.10 (USA), 0.4 (Serbia), 0.69 (Spain), 0.023 (Spain)	[16, 24, 33, 47]
	Amoxicillin	0.12 (Spain), 2.69–31.71 (*Tanzania*)	[36, 47]
Antimalaria	Artemether Lumefantrine	No immediate report as this medication is mainly used in Tanzania	Not reported
Hormone replacement	Estrone	0.0004 (USA), 0.0001–0.00157 (France), 0–0.67 (Ecuador), 0.0001–0.017 (USA)	[3, 7, 18, 19]
	Progesterone	1.0 (USA), 3.1 (USA), 0.005 (USA)	[1, 16, 19, 46]
*β*-blocker for treating abnormal heart rhythms	Atenolol	0.4 (Spain), 0.036 (USA), 0.86 (USA), 1.872 (Spain), 0.026 (USA)	[18, 39, 46, 47]
Estrogen, endocrine disrupting hormones	Estradiol	0.017 (USA), 0.0014–0.002 (Netherlands), 0–0.670 (Ecuador), 0.0002 (USA)	[3, 19, 46, 49]
	Estriol	0.0004 (USA), 0.0049–0.0121 (France), 0.005 (USA), 0.005 (USA)	[7, 16, 19, 34]
